# Biocompatible Bacterial Cellulose-Poly(2-hydroxyethyl methacrylate) Nanocomposite Films

**DOI:** 10.1155/2013/698141

**Published:** 2013-09-15

**Authors:** Andrea G. P. R. Figueiredo, Ana R. P. Figueiredo, Ana Alonso-Varona, Susana C. M. Fernandes, Teodoro Palomares, Eva Rubio-Azpeitia, Ana Barros-Timmons, Armando J. D. Silvestre, Carlos Pascoal Neto, Carmen S. R. Freire

**Affiliations:** ^1^Department of Chemistry and CICECO, Campus de Santiago, University of Aveiro, 3810-193 Aveiro, Portugal; ^2^Department of Cellular Biology and Histology, Faculty of Medicine and Odontology, University of the Basque Country (UPV/EHU), Barrio Sarriena s/n, 48940 Leioa, Spain; ^3^“Materials + Technologies” Group, Department of Chemical and Environmental Engineering, Polytechnic School, University of the Basque Country (UPV/EHU), Plaza Europa 1, 20018 San Sebastián, Spain

## Abstract

A series of bacterial cellulose-poly(2-hydroxyethyl methacrylate) nanocomposite films was prepared by *in situ* radical polymerization of 2-hydroxyethyl methacrylate (HEMA), using variable amounts of poly(ethylene glycol) diacrylate (PEGDA) as cross-linker. Thin films were obtained, and their physical, chemical, thermal, and mechanical properties were evaluated. The films showed improved translucency compared to BC and enhanced thermal stability and mechanical performance when compared to poly(2-hydroxyethyl methacrylate) (PHEMA). Finally, BC/PHEMA nanocomposites proved to be nontoxic to human adipose-derived mesenchymal stem cells (ADSCs) and thus are pointed as potential dry dressings for biomedical applications.

## 1. Introduction

Cellulose, the most abundant natural polymer, possesses unique properties and advantages [1–4], which have been widely explored for centuries, especially for paper making and for textile materials. More recently, cellulose fibres have also gained considerable and increasing attention as reinforcing elements in polymeric (nano)composite materials [1, 5–7]. Bacterial cellulose (BC) is a unique form of cellulose, produced by several bacteria of the *Gluconacetobacter* and *Sarcina* genus, among others [8, 9]. Because of its inherent biocompatibility and unique properties, that arise from the tridimensional network of nano- and microfibrils, bacterial cellulose is becoming a promising biopolymer for several biomedical [6, 10–16] (e.g., wound dressing, artificial skin, and scaffolds for tissue engineering and soft tissue replacement) and technological [17–21] applications (e.g., optical transparent nanocomposites, electronic paper, and fuel cell membranes). BC/polymer nanocomposites have been prepared by simple blending of BC nanofibrils with several polymeric matrices [22–27] or by *in situ* polymerization of monomers within the cellulose network [16, 28–32]. The latter approach is particularly straightforward because the properties of the nanocomposites can be easily tailored by adjusting the ratio of monomer/BC, the type and functionalities of the monomers, degree of cross-linking, and so forth. A limited number of monomers with acrylic/methacrylic moieties, such as glycerol monomethacrylate (GMMA) [16], 2-hydroxyethyl methacrylate (HEMA) [16, 31], 2-ethoxyethyl methacrylate (EOEMA) [16], acrylamide [28, 30], acrylic acid [29, 31, 32], and 2-ethylhexyl acrylate [31], have already been explored in this context, in particular for the development of BC/based hydrogels.

Poly(2-hydroxyethyl methacrylate) (PHEMA) is a versatile synthetic polymer with properties suited to a range of applications, in particular biomedical applications, including soft contact lenses [33], artificial corneas [34], degradable scaffolds for tissue engineering [35], and drug delivery systems [36]. BC/PHEMA hydrogels have already been described as part of two studies dealing with the preparation of BC based hydrogels by *in situ* polymerization of several acrylic monomers. In both cases the authors focus essentially on the swelling behaviour, morphology, and mechanical properties of the hydrogels. Other important properties, such as thermal stability, transparency, crystallinity, and biocompatibility, as well as their preparation in other forms such as films or aerogels, were not investigated and are also important for several applications. 

**Figure 1:**
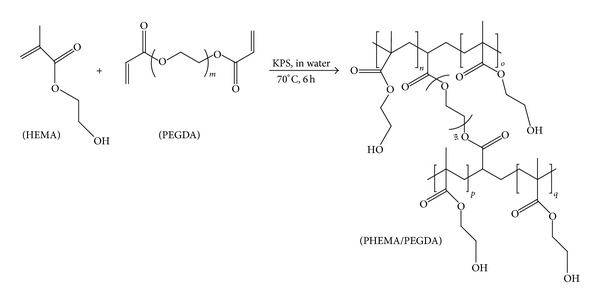
Schematic representation of HEMA polymerization, in the presence of PEGDA, to yield PHEMA cross-linked with PEGDA.

In the present study, BC/PHEMA nanocomposites in the form of thin films were prepared by *in situ* radical polymerization in the presence of poly(ethylene glycol) diacrylate (PEGDA) as cross-linker (Figure 1). The effect of the content of monomer and cross-linker was evaluated. The ensuing nanocomposites were characterized in terms of chemical structure, crystallinity, transparency, morphology, thermal stability, mechanical properties, and biocompatibility.

## 2. Materials and Methods

### 2.1. Chemicals and Materials

2-Hydroxyethyl methacrylate ((HEMA) 97%, stabilized) and poly(ethylene glycol) diacrylate ((PEGDA) average Mn 258, stabilized) were purchased from Sigma-Aldrich and used as received. Potassium persulfate ((KPS) 98%, Panreac) was used as thermal initiator. All other reagents and solvents were of analytical grade and used as received.

Bacterial cellulose (BC) (tridimensional network of nano- and microfibrils with 10–200 nm width) in the form of wet membranes was produced in our laboratory using the *Gluconacetobacter sacchari* bacterial strain [8] and following established procedures [37]. 

### 2.2. BC/PHEMA Nanocomposites Preparation

Wet BC membranes (~100 mg dry weigh, 4 × 4 cm^2^, and 0.8 cm thickness) were weighted, and 60% of their water content was removed with absorbent paper. Drained BC membranes were put in Erlenmeyers stopped with rubber septa and then purged with nitrogen. At the same time, aqueous solutions (5 mL) with different amounts of monomer HEMA (75; 150; 300 mg), 1.2% of KPS inititaor (w/w relative to monomer), and PEGDA (0, 1, and 5% (w_cross-linker_/w_monomer_)) were prepared (Table 1) and also purged with nitrogen (in an ice bath) for 30 min. Then, the solutions were transferred with a syringe to the Erlenmeyers containing the drained BC membranes. After that, the membranes were left to stand for 1 hour at room temperature (25°C) until the complete absorption/incorporation of the solutions. Subsequently, the reaction mixtures were left at 70°C (to induce *in situ* radical polymerization), for 6 h. Then, the septum was pulled off and the BC membranes washed with water (100 mL) during 30 min. This washing procedure was repeated three times. The washed membranes were placed over Petri dishes and dried at 40°C in a ventilated oven for 5–12 h. The dried membranes were kept in a desiccator until their use. All experiments were made in triplicate and analysed in the form of thin nanocomposite films. Samples of PHEMA and PHEMA cross-linked with PEGDA were prepared under the same conditions, in the absence of BC for comparative purposes.

Three samples from each series were freeze-dried and weighed. PHEMA/PEGDA and BC percent composition of the nanocomposites (Table 1) were estimated by difference to the original BC weight.

### 2.3. Nanocomposite Films Characterization

All ensuing films were characterized in terms of structure (FTIR and ^13^C NMR), morphology (SEM), crystallinity (XRD), transparency/opacity (visible light), thermal stability and degradation profile (TGA), thermodynamical properties (DMA), swelling behaviour, and biocompatibility.

FTIR spectra were taken with a Perkin-Elmer FT-IR System Spectrum BX spectrophotometer equipped with a single horizontal Golden Gate ATR cell over the range 600–4000 cm^−1^ at a resolution of 4 cm^−1^ averaged over 32 scans.

CPMAS ^13^C NMR spectra were recorded on a Bruker Avance III 400 spectrometer operating at a B0 field of 9.4 T using 9 kHz MAS with proton 90° pulse of 3 *μ*s. CPMAS ^13^C NMR spectra were acquired using a contact time of 2000 ms and a time between scans of 3 s. ^13^C chemical shifts were referenced with respect to glycine (C=O at 176.03 ppm).

SEM micrographs of the nanocomposite film surfaces were obtained on an HR-FESEM SU-70 Hitachi equipment operating at 1.5 kV and that of BC was taken with a Hitachi S4100 equipment operating in the field emission mode. Samples were deposited on a steel plate and coated with carbon.

The X-ray diffraction (XRD) measurements were carried out with a Phillips X'pert MPD diffractometer using Cu K*α* radiation.

The transmittance spectra of the nanocomposite films were collected with a UV-vis Spectrophotometer (Perkin-Elmer UV 850) equipped with a 15 cm diameter integrating sphere bearing the holder in the horizontal position. Spectra were recorded at room temperature in steps of 1 nm, in the range 400–700 nm.

TGA essays were carried out using a Shimadzu TGA 50 analyser equipped with a platinum cell. Samples were heated at a constant rate of 10°C/min from room temperature to 800°C under a nitrogen flow of 20 mL/min. The thermal decomposition temperature was taken as the onset of significant (~0.5%) weight loss, after the initial moisture loss.

Dynamic mechanical analyses (DMA) were performed on a Tritec 2000 DMA (Triton Technologies) using tension as deformation mode (single strain). For the temperature sweeps, a ramp rate of 2°C/min was used, and samples were heated from −100 to 200°C, at a frequency of  1 and 10 Hz, with a displacement of 0.005 mm. Samples' average dimensions of the films were approximately 5 × 5 × 0.2 mm. Tg values were determined using the maximum of the tan *δ* curve. Films used for DMA tests were kept in a conditioning cabinet at 50% relative humidity (RH) and 30°C to ensure the stabilization of their water content. PHEMA polymers (also previously conditioned) were analysed using the material pocket accessory in deformation mode.

The swelling ratio (SR) of the nanocomposite films was measured using the weighing method [5]. Specimens (dimensions 1 × 1 cm) were immersed in distilled water at room temperature to study their swelling in a minimum of three samples (tested for each material). The weight increase was periodically assessed for 2 days. Samples were taken out of the water; their wet surfaces were immediately wiped dry in filter paper and then reimmersed. Then the SR was calculated using equation (1)

(1) $$\begin{array}{c} \text{SR}\left(\%\right)=\frac{\left(W_{s}-W_{d}\right)}{W_{d}}\times 100\%, \end{array}$$

where *W*_*d*_ is the initial weight of dry film and *W*_*s*_ is the weight of the film swollen in water.

### 2.4. *In Vitro* Cell Response

Adipose-derived stem cells (ADSCs) were used in a short-term standard cytotoxicity assay of BC and BC/PHEMA/PEGDA (1 : 3 : 0.05) membranes. The procedure and methods are described elsewhere [38]. Briefly, membranes (6 cm^2^ of area, ≤0.5 mm thick) were sterilized with 70% ethanol for 2 h at room temperature and rinsed with phosphate buffered saline (PBS) aqueous solutions for 1 h. To prepare extracts of test materials according to the international standard ISO 10993-12, sterilized samples were incubated in ADSCs growth medium, consisting in Dulbecco's modified Eagle's medium ((DMEM) Sigma Chemicals Co., USA), supplemented with Glutamax (Sigma) and 10% fetal bovine serum ((FBS) Gibco), at 37°C for 24 h. The ratio of material surface/extract fluid was constant and equal to 6 cm^2^/mL.

For* in vitro *cytotoxicity assays, ADSCs were seeded and allowed to grow for 24 h in 96-well microplates at a density of 4 × 10^3^ cells/well in the presence of standard culture medium. Then, cultures were treated for 24, 48, and 72 h with the previously prepared extracted media. In addition, high-density polyethylene (negative control, USP Rockville, USA) and poly(vinyl chloride) (positive control, Portex, UK) were used. The metabolic activity of viable cells was determined by a colorimetric assay (Cell Proliferation Kit I MTT, Roche). Briefly, only viable cells could reduce MTT to formazan pigment, which is then dissolved in dimethylsulfoxide (DMSO). The cell number per well is proportional to the recorded absorbance of formazan at 550 nm, using an ELISA microplate reader. All assays were conducted in triplicate, and each experiment was repeated three times. Mean values and their standard deviations were calculated.

To analyze ADSCs seeding and proliferation onto the films, scanning electron microscopy (SEM) studies were carried out on cultured human ADSCs on BC and BC/PHEMA/PEGDA (1 : 3 : 0.05) membranes. Aliquots containing 5 × 10^4^ cells were seeded, under static conditions, onto BC and BC/PHEMA/PEGDA (1 : 3 : 0.05) membranes in ultralow attachment 24-well culture plates (Costar) pre-wetted with standard culture medium. The cultures were incubated for 72 h to allow attachment and proliferation (37°C, 5% CO_2_, and 95% RH). Subsequently, samples were rinsed with PBS to remove nonattached cells, fixed with 2% glutaraldehyde in a cacodylate buffer (0.1 M, pH = 7.4) and postfixed using OsO_4_ for 1 h, washed in phosphate buffer solution, and dehydrated using series of graded ethanol solutions. Samples were dried through CO_2_ critical point, gold sputtered, and analyzed in a SA-3400N Hitachi microscope. The voltage used was 15.0 kV, and magnifications selected for SEM images range from 1 500 to 10 000x.

## 3. Results and Discussion

**Figure 2:**
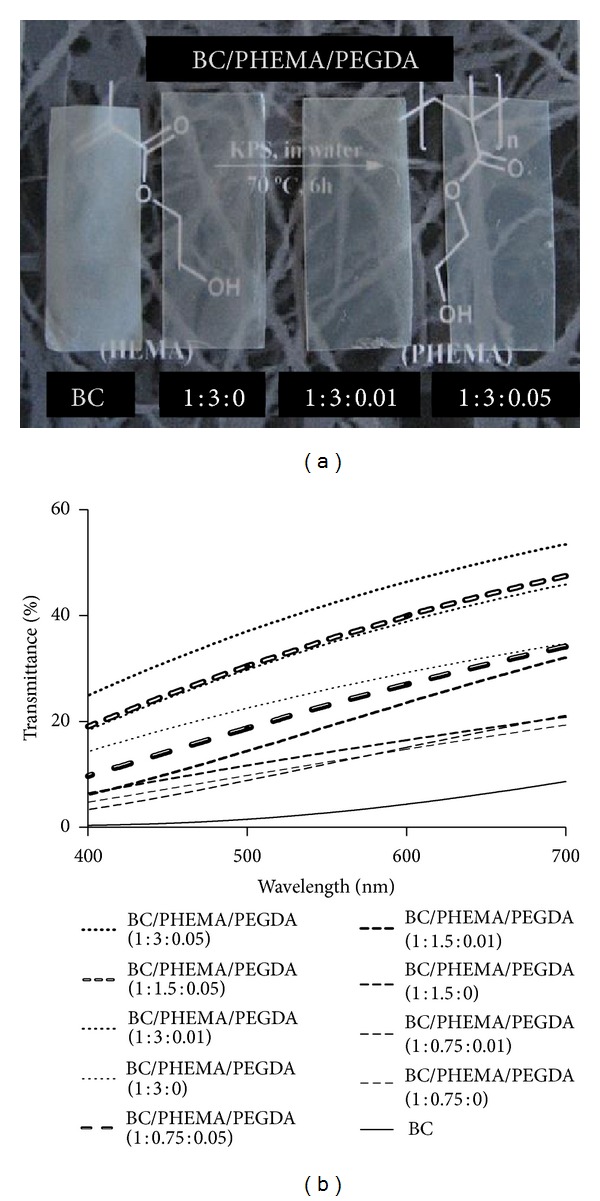
(a) Visual aspect and (b) transmittance of the different nanocomposite films.

A series of BC/PHEMA nanocomposite films was prepared by varying the amounts of monomer (HEMA) and cross-linker (PEGDA) impregnated into the BC membranes prior to the polymerization step (Table 1). All obtained nanocomposite films were very homogeneous (Figure 2(a)) and considerably more translucent than the pristine BC membrane. The amount of polymer imbibed in BC was shown to systematically grow with the amount of HEMA added as well as with the amount of cross-linker (PEGDA). In this last case, cross-linking will prevent PHEMA to be drained from BC network during washing or afterwards during any application of the material.

Figure 2(b) shows the light transmittance of all BC/PHEMA nanocomposite films, in the range of 400–700 nm. The incorporation of PHEMA polymeric chains into the BC nanofibrils network increases considerably the transmittance of the films, because of the inherent high transparency of this polymer [39] and of its excellent compatibility with the BC nanofibrils. The translucency increases with the amount of monomer and for the same monomer content increases also with the cross-linker content, confirming the expected higher retention of PHEMA inside the BC network promoted by the cross-linking of the polymeric chains.

### 3.1. Structural Characterization

The success of the polymerization reaction inside BC membranes was confirmed by FTIR and NMR analyses.

**Figure 3:**
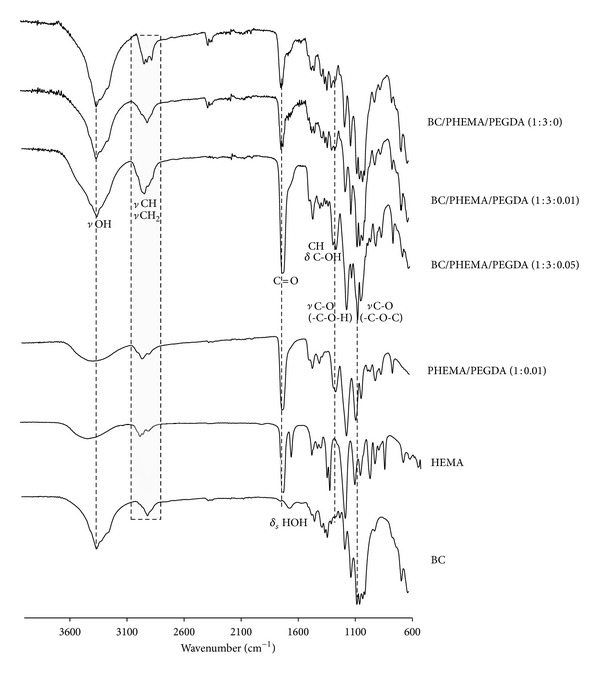
FTIR spectra of  BC, HEMA, PHEMA/PEGDA (1 : 0.01), and BC/PHEMA nanocomposite films (1 : 3).

Figure 3 shows the FTIR spectra of BC, HEMA, and PHEMA (prepared in the same conditions but without cellulose and cross-linker) and of BC/PHEMA nanocomposites with higher content of PHEMA as an example (series BC/PHEMA/PEGDA (1 : 3)). All BC/PHEMA films produced by this methodology present, as expected, an FTIR spectral trace correspondent to the sum of both components (BC and PHEMA). The success of the polymerization of HEMA inside the BC membranes was confirmed by the appearance in the films of an intense band at around 1716 cm^−1^, attributed to the carbonyl ester group stretching vibrations in the polymer and to the concomitant disappearance of the band at 1634 cm^−1^ and of the sharp peak at 814 cm^−1^, assigned, respectively, to the C=C stretching vibration and to C–H out-of-the-plane bending vibration, from the vinyl group of the monomer. The bands at 1447 and 747 cm^−1^, associated with the bending vibration mode of CH_2_ and CH_2_ rocking, characteristic of methacrylic polymers are also a confirmation of the successful formation of PHEMA inside the BC network. Finally, the vibrations at 3350 cm^−1^ (O–H stretching), 2930 cm^−1^ (C–H stretching), and 1245 cm^−1^ (stretching of C–O) are typical of both BC and PHEMA. Moreover, the comparison of the normalized FTIR spectra of the BC/PHEMA/PEGDA (1 : 3) nanocomposite series clearly confirmed that the amount of PHEMA retained inside the BC network increased with the cross-linker content based on the increment of the intensity of the characteristic bands of PHEMA, namely, the carbonyl ester stretching vibrations at around 1715 cm^−1^. This tendency was also observed for the other series (BC/PHEMA/PEGDA (1 : 1.5) and BC/PHEMA/PEGDA (1 : 0.75)) and is in close agreement with the transmittance results described above. 

**Figure 4:**
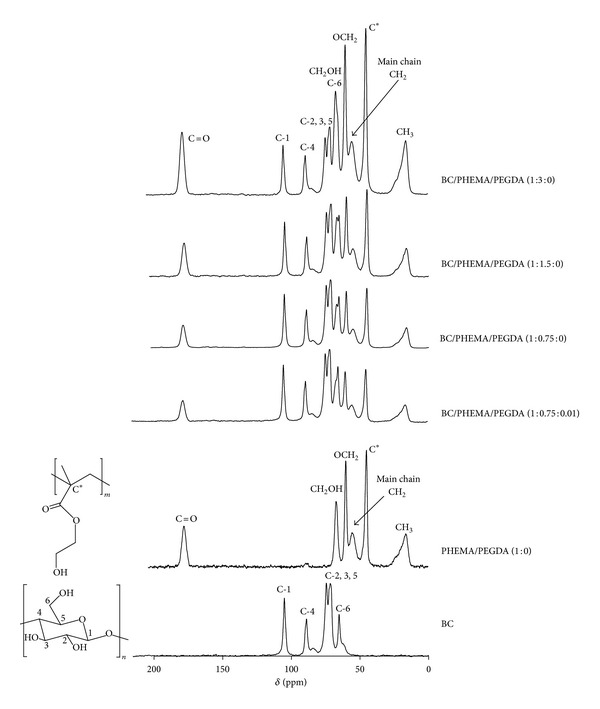
CPMAS ^13^C NMR spectra of nanocomposite films, BC/PHEMA/PEGDA (1 : 3 : 0), (1 : 1.5 : 0), and (1 : 0.75 : 0), BC/PHEMA/PEGDA (1 : 0.75 : 0.01) and of PHEMA/PEGDA (1 : 0) and BC.

Figure 4 displays the solid state CPMAS ^13^C NMR spectra of selected BC/PHEMA nanocomposite films, namely, BC/PHEMA/PEGDA (1 : 3 : 0), BC/PHEMA/PEGDA (1 : 1.5 : 0), BC/PHEMA/PEGDA (1 : 0.75 : 0), and BC/PHEMA/PEGDA (1 : 0.75 : 0.01) as well as of starting components, BC, and PHEMA. It is evident that, the ^13^C NMR spectra of the BC/PHEMA nanocomposites are also a sum of the resonances typical of BC carbons at *δ* 65.2 (C-6), 71.4–74.3 (C-2,3,5), 90.0 (C-4), and 104.8 ppm (C-1) and of PHEMA at 16.2-23.2 (*α*-CH_3_), 45.0 (quaternary C), 55.4 (CH_2_ main chain), 60.1 (–O–CH_2_), 67.2 (HO–CH_2_–), and finally 178.2 ppm assigned to C=O. Carbon resonances specific of PEGDA are not visible due to the low content of this last component. The intensity of the PHEMA carbon resonances compared to those of BC increases with the proportion of monomer and cross-linker (data not shown) used in each experiment, reflecting different polymer contents, which is in good agreement with the FTIR analysis and the weight gains reported above (Table 1).

The absence of FTIR vibrations or ^13^C resonances typical of the monomer (or of other contaminants) in the FTIR and NMR spectra of the nanocomposites demonstrates the complete consumption/removal of all reactants/byproducts during the polymerization and washing steps which is a crucial aspect when considering biomedical applications.

### 3.2. Morphology Characterization

**Figure 5:**
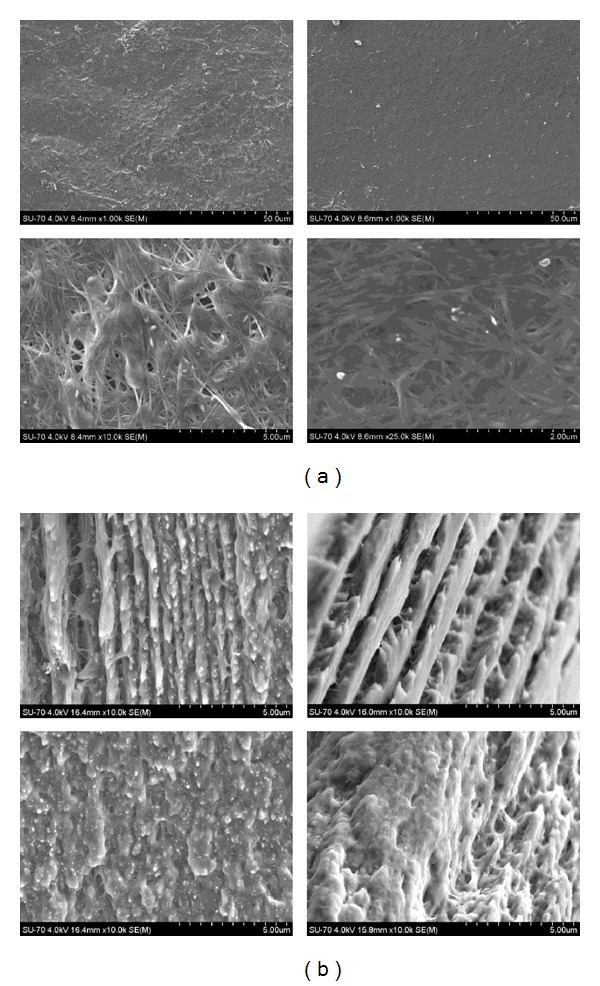
SEM micrographs of BC/PHEMA/PEGDA (1 : 3 : 0) (left) and BC/PHEMA/PEGDA (1 : 3 : 0.05) (right) nanocomposite films, recorded from surfaces (a) and cross-sections (b).

A selection of SEM micrographs of the surface of BC/PHEMA (1 : 3) nanocomposites with 0% and 5% of cross-linker is shown in Figure 5(a).

The characteristic tridimensional nanofibrillar network of BC [24, 38] was clearly observed in the surface of all nanocomposite films indicating that this was not affected by the polymerization reaction. In addition, the cellulose nanofibrils are perfectly embedded within the PHEMA matrix. The nanocomposites without cross-linker displayed a less homogenous morphology, with several unfilled parts, suggesting a considerable superficial lixiviation of PHEMA during the washing step.

For the same nanocomposite films a selection of SEM micrographs of the fractured zones (cross-section) is shown in Figure 5(b). For each sample, two different magnifications were used in order to display the distribution of PHEMA in the BC network and the interfacial adhesion between the two composite components. The cross-section micrographs of both nanocomposite films displayed the typical lamellar morphology of BC completely impregnated with PHEMA. These images also provided evidence of the strong interfacial adhesion between BC nanofibrils and PHEMA, as shown by the nanofibers breakage during fracturing and the homogeneous dispersion of the matrix within the BC network. This behaviour is obviously due to the excellent compatibility between BC and PHEMA that arises from the potential establishment of hydrogen bonds between them. These results clearly support the superior mechanical properties of the BC/PHEMA nanocomposites, as suggested by the mechanical tests discussed below.

### 3.3. X-Ray Diffraction Characterization

**Figure 6:**
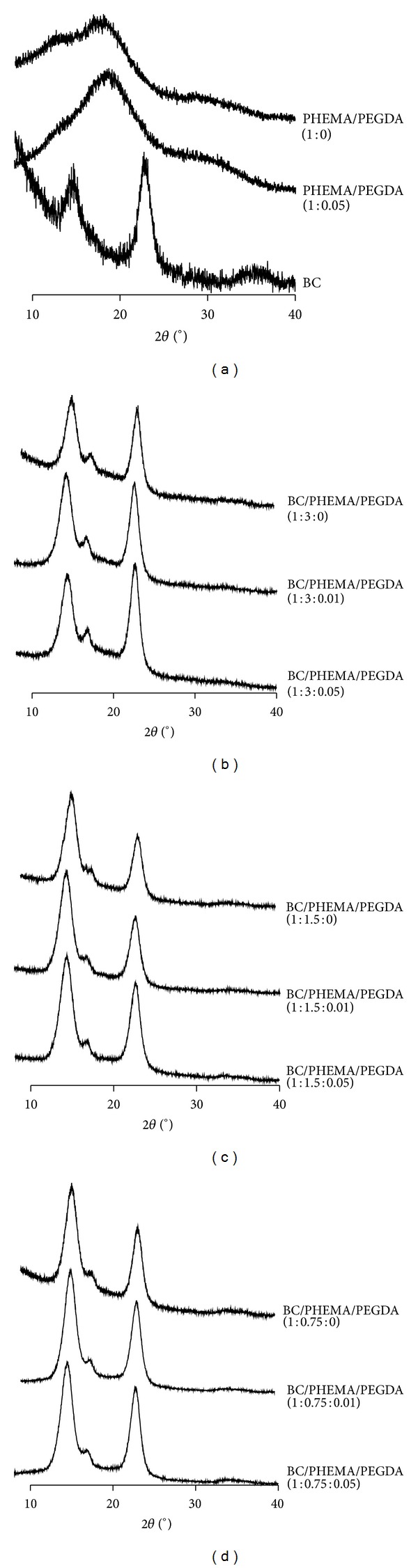
X-ray diffractograms of the nanocomposite films, polymers (with and without cross-linker), and BC.

X-ray diffraction analyses have been performed on neat BC membranes, PHEMA matrices (with different amounts of cross-linker), and all BC/PHEMA nanocomposite films (Figure 6). 

As it is well known, BC exhibits a diffractogram typical of Cellulose I (native cellulose), with the main diffraction peaks at 2**θ** 14.3, 15.9, 22.6, and 33.7° [40], while all PHEMA matrices are characterized by a broad peak centred at around 2**θ** 18°, typical of fully amorphous materials. The X-ray diffraction profiles of the BC/PHEMA nanocomposite films only showed that the typical diffraction peaks of BC and their magnitude increases, as expected, with the BC content. For the nanocomposite films with lower cross-linker content these peaks are quite evident. In fact, the diffractograms of these materials look very closely to that of BC. The increase on crystallinity observed for the nanocomposite films, together with the unique BC morphology, is closely related to the improvement of the mechanical performance of the PHEMA/BC based films discussed later.

### 3.4. Swelling Behaviour

**Figure 7:**
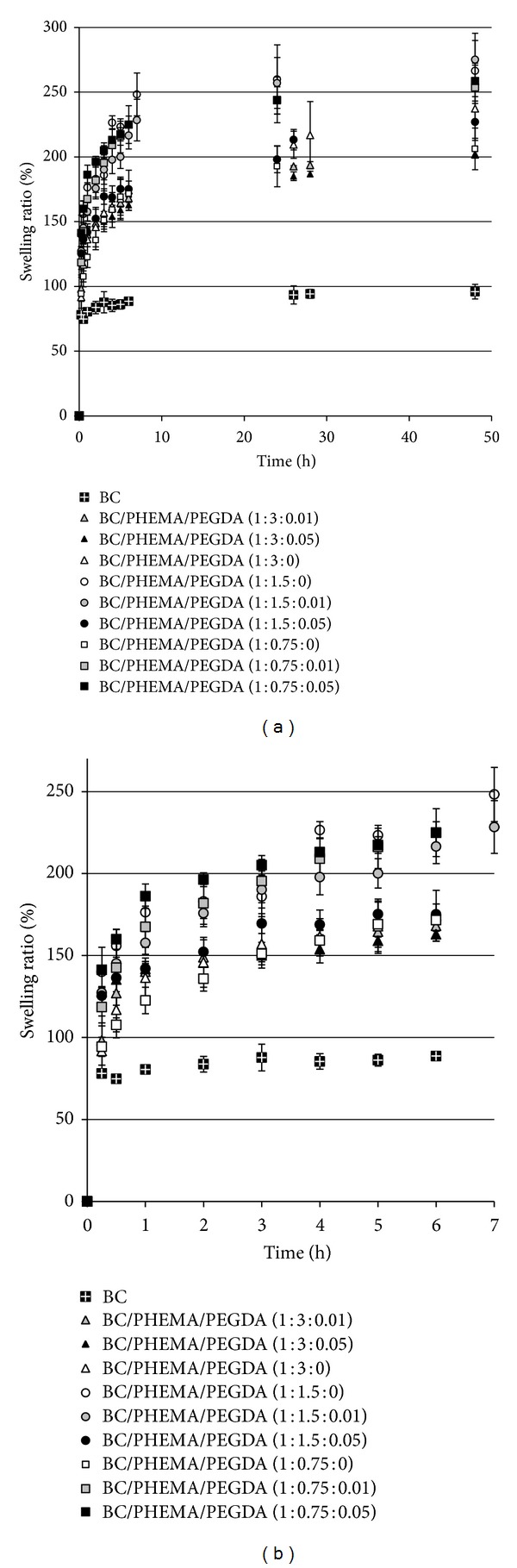
(a) Plot of the swelling ratio as a function of time of all BC/PHEMA nanocomposite films and BC membrane (0–48 h). (b) Expansion 0–7 h.

Swelling studies were performed in order to evaluate the rehydration ability of the nanocomposite films by their immersion in water during at least 48 h (reverse swelling after drying). Swelling ratios of the native BC membrane (for comparison) and BC/PHEMA nanocomposite films are presented in Figure 7(a) (0–48 h) and Figure 7(b) (expansion, 0–7 h). All samples absorbed water during the experiment, following similar patterns. After a relatively fast water uptake during the first hour, the water absorption slowed down, leading gradually to a plateau after 24 hours. However, the BC/PHEMA nanocomposite films showed a considerably higher swelling ratio than BC membranes, specifically 200–270% and 100%, respectively. This behaviour is attributed to the presence of the hydrophilic PHEMA polymeric chains within the nanostructured BC network which additionally prevents the collapse of BC nanostructure during drying. 

The PHEMA content in the nanocomposites, that is, the amount of PHEMA retained in the BC network, is strictly related to the improvement on their swelling ratio being in general higher for those with cross-linker, which is in close agreement with the NMR and FTIR analyses. These nanocomposites increase the water retention capacity of BC, in its film form, originating improved BC nanocomposite films suitable for several biomedical applications, such as, wound healing and topical drug delivery.

### 3.5. Thermal Properties

**Figure 8:**
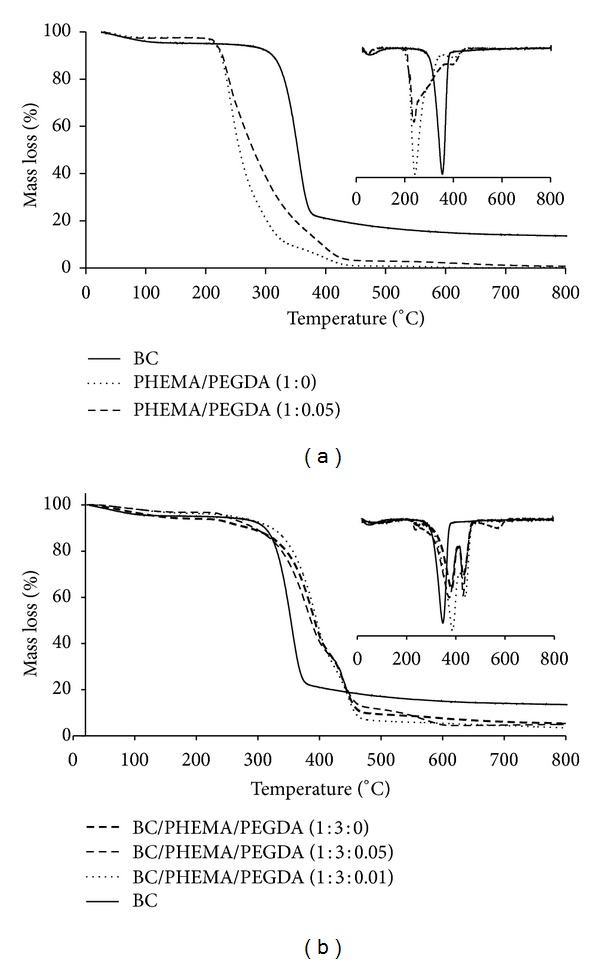
(a) TGA thermograms of BC and PHEMA/PEGDA (1 : 0) and PHEMA/PEGDA (1 : 0.05). (b) TGA thermograms of BC/PHEMA/PEGDA (1 : 3 : 0), BC/PHEMA/PEGDA (1 : 3 : 0.01), and BC/PHEMA/PEGDA (1 : 3 : 0.05).

Thermogravimetric analysis of BC/PHEMA nanocomposite films was carried out to evaluate their thermal stability and degradation profile (Table 2, Figure 8). The thermal stability is a quite important aspect in several applications where materials might be submitted to high temperatures, such as sterilization in the case of biomedical materials. Reference BC membrane and PHEMA matrices (with and without cross-linker) were also analysed for comparison purposes (Figure 8).

BC showed a single weight-loss feature, typical of cellulosic substrates, with initial and maximum decomposition temperatures at around 260 and 350°C, respectively [41]. The mass loss at around 100°C is associated with the volatilization of residual water. PHEMA matrices are considerably less stable than BC since they start to decompose at around 200°C and presented a degradation profile with three main degradation steps at about 230, 290, and 400°C (Figure 8) [42]. The cross-linking of PHEMA with small amounts of PEGDA (up to 5%) had no measurable effect on the thermal stability of the polymer.

The TGA tracing of BC/PHEMA nanocomposite films is not a sum of those of the individual components since they showed in general a two-step weight-loss degradation profile with maximum degradation temperatures at 370–390 and 430–440°C (Table 2, Figure 8). The distinct degradation profiles and the considerable increments on the Ti and Tdmax of the nanocomposites when compared with BC and PHEMA clearly suggest a strong interaction between them and thus excellent compatibility, as previously observed by SEM. This is probably associated with the establishment of strong interactions (hydrogen bonds) between BC nanofibrils and PHEMA chains. The range of PHEMA percentage in the nanocomposites studied here hardly had any effect on the thermal stability and degradation profile of the films.

### 3.6. Dynamic Mechanical Properties

**Figure 9:**
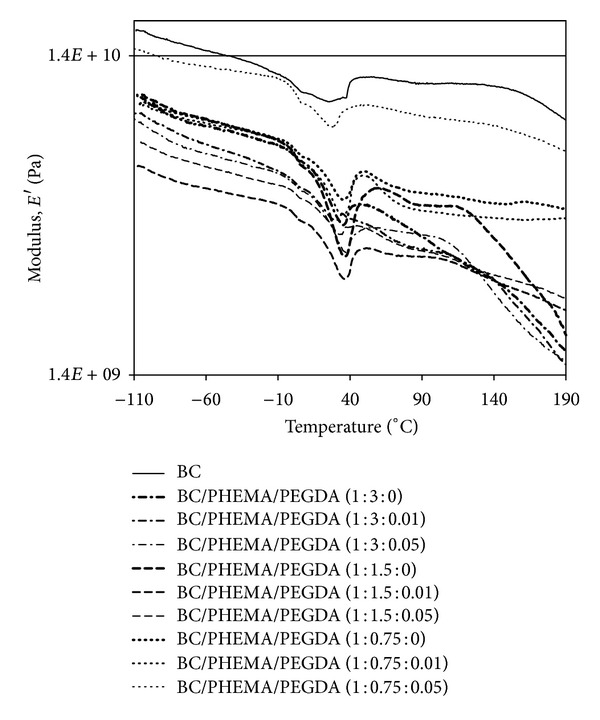
*Storage moduli versus* temperature of BC and all nanocomposite films. DMA analyses were carried out in tension mode (1 Hz). All the samples were conditioned at 50% RH.

Figure 9 shows the variation of the *storage tensile modulus* of BC and BC/PHEMA nanocomposite films as a function of temperature. Specifically, the effect of the amount of PHEMA (and BC) and cross-linker on the viscoelastic properties of the nanocomposites was assessed.

For neat BC membranes the variation of  *E*′ as a function of temperature only showed a minor transition at around 40°C attributed to the release of residual water molecules which are known to act as plasticizers. This transition was also observed in all BC/PHEMA nanocomposites since PHEMA is also considerably hydrophilic. For the nanocomposite films with higher PHEMA contents (Table 1), an additional transition was registered in the range 100–160°C, with a maximum decrease at around 125°C. This drastic drop of the tensile *storage modulus* *E*′ is typical of a relaxation phenomenon associated with the glass-rubber transition of the PHEMA matrix [43], as further confirmed by the analysis of neat PHEMA.

As expected all nanocomposites showed lower *storage moduli* than that of neat BC because PHEMA matrices are amorphous and thus less rigid than the BC membrane which is a highly crystalline material. These results clearly indicate that a set of BC/PHEMA nanocomposite films with distinct mechanical performances (but similar thermal stabilities) can be easily designed by simply varying the BC/PHEMA percentage contents as well as the percentage of cross-linker. Thus this strategy consists in a simple approach to obtain PHEMA based nanocomposite films for applications requiring diverse mechanical performances.

### 3.7. Biocompatibility

The effect of the introduction of PHEMA polymeric chains into the BC membrane network on the viability, proliferation, and cell adhesion of human ADSCs was studied. A wide variety of cell lines have been recently used to determine cytotoxicity and biocompatibility of novel materials based on BC, including ADSCs [44]. This type of cells has emerged as an important tool for tissue engineering because they exhibit capacity to differentiate into mesodermal cell lineages, largely to bone, cartilage, and adipocytes [45, 46]. 

**Figure 10:**
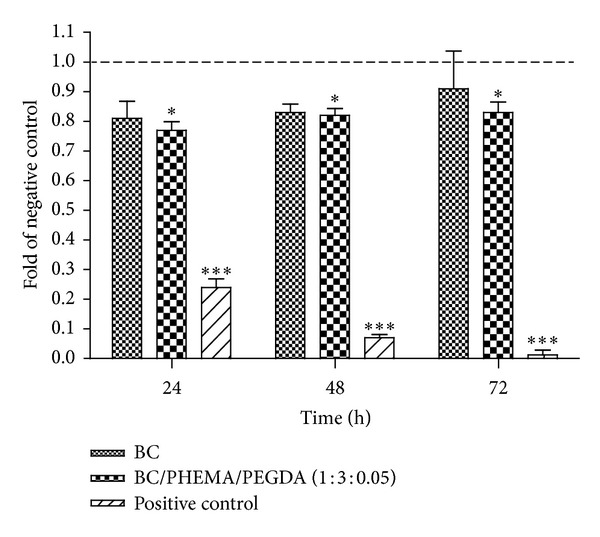
ADSCs proliferation in contact with BC, BC/PHEMA/PEGDA (1 : 3 : 0.05) membranes, and positive and negative control during 24, 48, and 72 h. Data are presented as mean ± standard deviation of three independent experiments (*P < 0.05).

Figure 10 shows the cell viability based on the cell growth ratio between extracted fluid from samples and negative control. As expected, standard growth values were obtained with the negative control (high-density polyethylene (HDPE)) and a dramatic reduction of cell number was found with the positive control (poly(vinyl chloride) (PVC)). Regarding cells cultured with extracted media, obtained as described previously with BC and BC/PHEMA/PEGDA (1 : 3 : 0.05) membranes, no significant differences in proliferation rates were observed among them when we compare with negative control; whereas no significant differences exist in growth rate of cells cultured with BC extracted media, a slightly significant lower growth rate was found in the case of cells cultured in BC/PHEMA/PEGDA (1 : 3 : 0.05) extracted media (*P* < 0.05). However, considering that viability and proliferation rates above 70% of the control, specifically, 91% in BC, and 83% in BC/PHEMA/PEGDA (1 : 3 : 0.05) membranes were observed here and that according to EN ISO 10993-5:200960 a material is considered cytotoxic if cell viability is reduced by more than 30%, we can state that BC and BC/PHEMA/PEGDA (1 : 3 : 0.05) are not cytotoxic for ADSCs.

**Figure 11:**
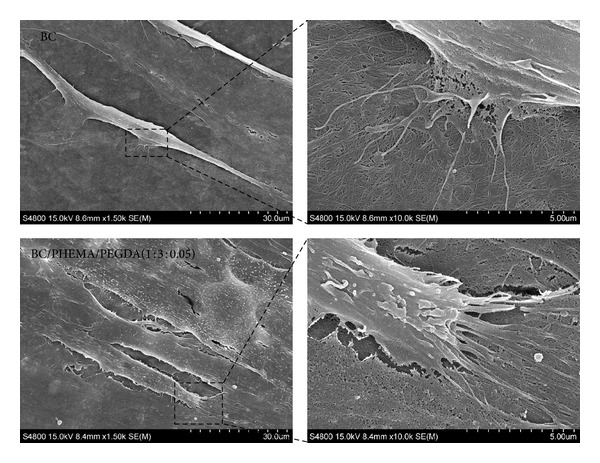
SEM images showing morphology of ADSCs at 72 h after seeding on the surface of BC and BC/PHEMA/PEGDA (1 : 3 : 0.05) membranes.

With respect to the ADSC seeding assessment, SEM analysis was conducted in order to determine cell morphology, spreading, and adhesion onto BC and BC/PHEMA/PEGDA (1 : 3 : 0.05) membranes.  Figure 11 shows the micrographs of ADSCs taken after 72 h of seeding on these membranes. In both membranes, ADSCs were well spread, adhered correctly, and proliferated to form a continuous layer of cells fully covering the membranes.

As shown in low magnification photomicrograph (1500x) ADSCs displayed a spindly morphology with numerous cytoplasmic projections firmly attached to material surfaces. In the enlarged images (10000x) the thin cytoplasmic projections adhere actively to the porous network and natural nanofibers structure of the materials. In fact, these nanostructured membranes seem to be ideal for harboring cell growth.

## 4. Conclusions

In the present work, a systematic study for the production of BC/PHEMA nanocomposite films was performed, via *in situ* radical polymerization of HEMA, using PEGDA as cross-linker, tailoring its properties by the simple ranging of the polymer and/or cross-link content in relation to BC content, while the BC nanostructure was retained. The obtained nanocomposite films were fully characterized in terms of composition, transparency, crystallinity, morphology, swelling, thermal and mechanical properties, cytotoxicity, and biocompatibility.

These films are translucent and are considerably more thermally stable than PHEMA matrix. These composites are less rigid materials when compared to BC, which is confirmed by a decrease in the *storage tensile modulus*, and present good swelling ratios (~200–260%). Biocompatibility studies demonstrated that BC/PHEMA nanocomposite films are noncytotoxic providing a favourable cell environment for optimal adhesion and proliferation of ADSCs. Because of this, BC/PHEMA can therefore be seen as a promising material for several biomedical applications, including the design of 3D matrices to maintain a cellular niche for stem cell-mediated tissue regeneration.

## Figures and Tables

**Table 1:** Identification of the nanocomposite films and component contents estimation.

BC/PHEMA/PEGDA nanocomposites	Dry BC (mg)	HEMA (mg)	PEGDA (%)	PHEMA (%)	BC (%)
BC/PHEMA/PEGDA (1 : 3 : 0)	100	300	0	74.0	26.0
BC/PHEMA/PEGDA (1 : 3 : 0.01)			1	73.9	26.1
BC/PHEMA/PEGDA (1 : 3 : 0.05)			5	75.9	24.1

BC/PHEMA/PEGDA (1 : 1.5 : 0)	100	150	0	57.0	43.0
BC/PHEMA/PEGDA (1 : 1.5 : 0.01)			1	59.2	40.8
BC/PHEMA/PEGDA (1 : 1.5 : 0.05)			5	61.0	39.0

BC/PHEMA/PEGDA (1 : 0.75 : 0)	100	75	0	36.1	63.9
BC/PHEMA/PEGDA (1 : 0.75 : 0.01)			1	40.3	59.7
BC/PHEMA/PEGDA (1 : 0.75 : 0.05)			5	44.8	55.2

**Table 2:** Thermal degradation profiles of the studied nanocomposite films.

BC/PHEMA nanocomposites	% mass loss at 100°C	Tdi (°C)	Tdmax1 (°C)	Tdmax2 (°C)
BC/PHEMA/PEGDA (1 : 3 : 0)	3.36	272	384	434
BC/PHEMA/PEGDA (1 : 3 : 0.01)	1.79	274	388	442
BC/PHEMA/PEGDA (1 : 3 : 0.05)	1.79	250	373	434
BC/PHEMA/PEGDA (1 : 1.5 : 0)	2.95	276	395	443
BC/PHEMA/PEGDA (1 : 1.5 : 0.01)	2.25	266	387	440
BC/PHEMA/PEGDA (1 : 1.5 : 0.05)	2.81	257	373	430
BC/PHEMA/PEGDA (1 : 0.75 : 0)	3.03	276	389	442
BC/PHEMA/PEGDA (1 : 0.75 : 0.01)	2.06	272	390	441
BC/PHEMA/PEGDA (1 : 0.75 : 0.05)	2.44	235	386	433
